# An Integrated Monolithic Synaptic Device for C-Tactile Afferent Perception and Robot Emotional Interaction

**DOI:** 10.34133/cbsystems.0367

**Published:** 2025-08-19

**Authors:** Yue Li, Lu Yang, Qianbo Yu, Yi Du, Ning Wu, Wentao Xu

**Affiliations:** ^1^ Institute of Photoelectronic Thin Film Devices and Technology, Key Laboratory of Photoelectronic Thin Film Devices and Technology of Tianjin, College of Electronic Information and Optical Engineering, Engineering Research Center of Thin Film Photoelectronic Technology of Ministry of Education, Academy for Advanced Interdisciplinary Studies, Nankai University, Tianjin 300350, China.; ^2^ Shenzhen Research Institute of Nankai University, Shenzhen 518083, China.

## Abstract

C-tactile afferents are low-threshold mechanoreceptors that innervate the hairy skin of mammals, essential for emotional interactions. Replication of such a mechanism could facilitate emotional interactions between humans and embodied intelligence robotic systems. Herein, we demonstrate a monolithic synaptic device that replicates and integrates tactile sensing and neuromorphic processing functions for in-sensor computing. The device is operable by both mechanical and electrical inputs, with the mechanoelectrical operation mechanism stemming from the synergistic effect of dynamic ionic migration and injection. As a proof of concept, the device effectively converts spatiotemporal tactile stimuli into distinct electrical signals, which are subsequently encoded to enable the microcomputer to classify multiple discrete emotional states, such as happiness, calmness, and excitement. This monolithic integrated device, which converges mild tactile perception with neuromorphic processing, with high tactile sensitivity and low-energy consumption, establishes an approach for emotional interaction between intelligent robots and human beings.

## Introduction

The human somatosensory system possesses a distributed architecture comprising diverse mechanoreceptors, e.g., Meissner corpuscles, and hierarchical neural networks that span peripheral to central pathways [1]. This bioinspired framework achieves efficient spatiotemporal integration through event-driven neuromorphic processing, where primary sensory signals undergo multistage feature refinement across synaptic layers [2–4]. In the tactile afferent system, C-tactile (CT) afferents represent a distinct class of low-threshold, unmyelinated mechanoreceptors that innervate mammalian hairy skin. These specialized nerves constitute a dedicated neurobiological pathway responsible for transducing gentle tactile stimuli into affective emotional states [5,6]. Notably, CT afferents play a crucial role in mediating interpersonal emotional interactions, acting as essential neurophysiological foundations for social communication.

The emergence of sensory neuromorphic devices enabling in-sensor computing has led to a paradigm-shifting innovation in intelligent sensing architectures, enabling signal transduction and parallel information processing through the physical integration of sensory and computational elements [7–9]. Neuromorphic visual systems implementing the bioinspired framework have achieved visual pattern recognition [10], motor behavior detection [11,12], and dynamic luminance adaptation [13], demonstrating remarkable potential. Unlike visual perception, tactile sensing fundamentally relies on direct physical contact between mechanical stimuli and sensory transducers. Most current tactile sensing remains constrained by conventional 3-stage architectures maintaining segregated sensation, transmission, and processing modules [14–16]. These discrete configurations inevitably suffer from systemic latency accumulation through serial signal conversion stages and energy-intensive analog-to-digital transduction cascades, critically impairing temporal fidelity in closed-loop haptic feedback systems.

Recent advances in tactile in-sensor computing have yielded artificial synaptic transistors with pressure-modulated gate dielectrics, demonstrating programmable mechanoelectronic coupling [17,18]. Different from traditional tactile sensors, tactile in-sensor computing requires direct use of force inputs to modulate synaptic weight, so that a relatively large force is often required to drive the computational processing function in such integrated devices [19,20]. Therefore, a monolithic device that emulates CT sensitive perception and processing is still desired. Moreover, critical translational challenges persist in transforming these devices into practical human–robotic interface systems. The universal deficiency in the biocompatibility of existing gate dielectrics fundamentally impedes epidermal integration and chronic implantation potential. Concurrently, these systems have neglected the implementation of multimodal tactile decoding visualization—a crucial requirement for closed-loop human–machine interaction and real-time tactile feedback systems.

**Fig. 1.**
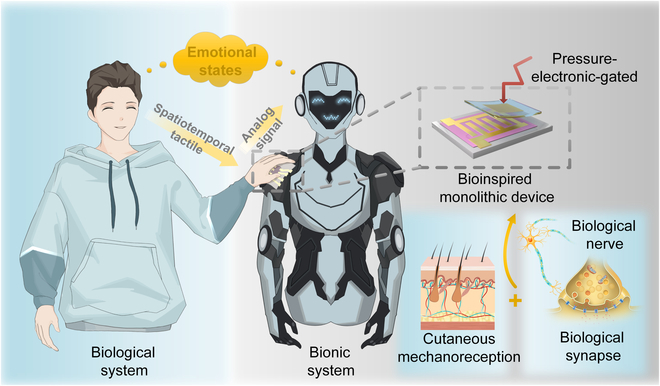
A pressure-mechanical integrated synaptic device inspired by human skin mechanoreceptors and synapses that recognizes different touch patterns for discrete affective state classification.

Herein, we demonstrate a monolithic pressure-electronic-gated (PEG) neuromorphic device that replicates CT afferents, for low-threshold mechanosensation and neuromorphic information processing in the same device (Fig. 1). Under synergistic pressure and electric field regulation, the device achieves real-time pressure sensing and information processing through dynamic ionic migration and injection process to modulate synaptic weights. Compared to previous integrated neuromorphic devices, our system is responsive to ultralow threshold pressures down to 80 Pa and demonstrates a broad operational range of almost 3 orders of magnitude (0.039 to 24.872 μA), operable at ultralow driving voltages (−0.2 V) (Table S1).

The architecture enables hardware-level integration of tactile sensing and neuromorphic processing. Particularly, it successfully decodes spatiotemporal tactile patterns, including contact pressure and stimulating frequency, into distinguishable electrical signals, which are interpretable by a microcomputer as discrete emotional states, such as happiness, calmness, and excitement. This work is potentially suitable for enhancing empathic communication in socially assistive robotics applications such as autism therapy and elderly care in the future.

## Methods

### Preparation of chitosan hydrogel

Chitosan powder (0.1 g, Sigma-Aldrich, ≥95% purity) was dissolved in 10 ml of acetic acid solution (2 wt %, Aladdin, ≥99.8% purity) with the addition of 0.2 ml of glycerol (plasticizer, Macklin). The mixture was stirred (600 rpm) at 70 °C for 2 h until the chitosan powder was dissolved. The solution was sonicated to remove air bubbles. The solution was applied evenly to the glass slide and thermally cured in a vacuum (10^−3^ torr) oven at 80 °C for 30 min, yielding free-standing chitosan hydrogel membranes.

### Preparation of the PEG synaptic device

A poly(3-hexylthiophene) (P3HT) solution (20 mg/ml, Sigma-Aldrich, regioregularity >98%) was prepared in anhydrous chloroform (Alfa Aesar, 99.8% purity), by magnetically stirring in a sealed vial at 60 °C for 4 h under a nitrogen atmosphere until achieving a clear solution. The clarified P3HT solution was spin-coated (1,500 rpm, 40 s) onto a SiO_2_–Si substrate (300-nm oxide layer) pretreated with UV ozone for 20 min to enhance surface hydrophilicity. Au source/drain electrodes (50 nm) were obtained through thermal evaporation. Chitosan hydrogel was precisely transferred onto active channels. Finally, an Au top gate electrode (50 nm) was deposited through thermal evaporation.

### Characterizations and measurements

Fourier transform infrared spectra were acquired with a Bruker VERTEX 70 spectrometer. Scanning electron microscopy and energy-dispersive x-ray spectroscopy were performed using an FEI Apreo S LoVac field-emission scanning electron microscope. Electrical characterization was performed using a Keithley 4200A-SCS parameter analyzer coupled with a probe station. Pressure-dependent tactile response measurements utilized Keithley 2400 SourceMeter and an LCR tester integrated with Mark-10 for capacitance change measurement at different pressures.

## Results and Discussion

### Bioinspired synaptic device

**Fig. 2.**
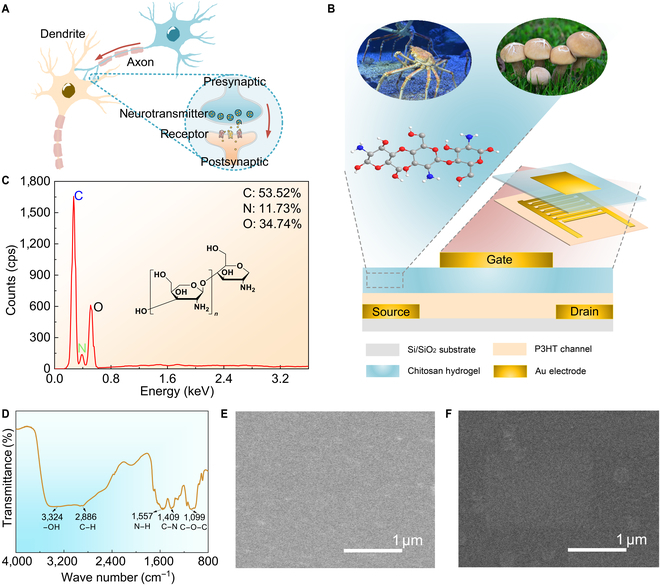
Structure and material characterization of the artificial synaptic device. (A) Biological neuron and synaptic structure. (B) Structure diagram of the artificial synaptic device. (C) Energy-dispersive x-ray spectroscopy (EDS) of the chitosan hydrogel. (D) Fourier transform infrared spectroscopy (FT-IR) of the chitosan hydrogel. (E and F) Scanning electron microscopy (SEM) images of the chitosan and poly(3-hexylthiophene) (P3HT) layers.

CT afferents as a specialized low-threshold mechanoreceptors include a dual-component architecture: low-threshold mechanosensitive units and neuroinformatic transmission systems [5,6]. Synapses interconnect neurons to form networks, underlying information transmission, processing, and memory (Fig. 2A) [21–23]. In afferent nerves, synapses filter unnecessary information and pass useful information to the next layer of neurons [24].

We fabricated a 3-terminal neuromorphic device that replicates C-afferent nerves for the mechanoperception of tactile information and signal transmission in the form of a postsynaptic current (PSC; Fig. 2B). The device architecture comprises a proton-conductive chitosan gate dielectric, a solution-processed P3HT semiconducting channel, and Au source/drain electrodes. This architecture emulates ionic migration in biological synapses, and the chitosan layer facilitates neurotransmitter-analogous transport through dynamic ionic diffusion, while P3HT enables ionic trapping/detrapping processes mimicking postsynaptic receptor activation.

Chitosan is a natural product from fungal cell walls, e.g., basidiomycetes, and crustacean exoskeletons, e.g., crabs [25,26], extensively utilized in biosensing [27,28], energy harvesting [29], and biodegradable textiles [30], showing exceptional biological compatibility and eco-sustainability. For device fabrication, we employed a standardized acetic acid solution for chitosan dissolution [31,32]. Elemental composition analysis by energy-dispersive x-ray spectroscopy revealed characteristic stoichiometric ratios of C (53.53), O (34.74), and N (11.73) (Fig. 2C). Fourier transform infrared spectroscopy further confirmed the functional groups in the polymer, e.g., –OH groups centering at ~3,324 cm^−1^, C–H stretching at ~2,923 cm^−1^, N–H bending deformation at ~1,557 cm^−1^, C–N stretching vibration at ~1,409 cm^−1^, and C–O–C stretching vibration at ~1,099 cm^−1^ (Fig. 2D). Scanning electron microscopy shows the smooth surfaces of both chitosan hydrogel and P3HT layers (Fig. 2E and F).

### Voltage-gated synaptic plasticity in the artificial synaptic device

**Fig. 3.**
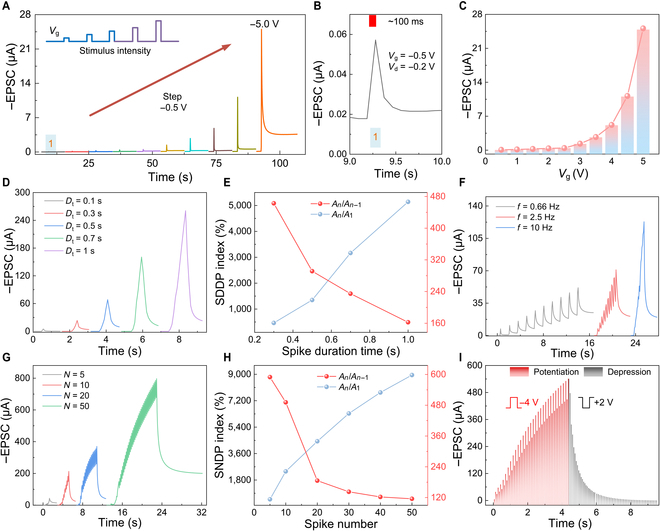
Testing of synaptic devices. (A) Excitatory postsynaptic current (EPSC) of the device under different pulse amplitudes (−0.5 to −5 V). (B) Magnification under −0.5 V. (C) Maximum EPSC of the device at different pulse voltages. (D and E) EPSC and change ratio under different pulse durations, *A_n_*/*A*_*n*−1_ and *A*_*n*_/*A*_1_ of the *n* pulse to the previous pulse. (F) EPSC of devices at different pulse frequencies. (G and H) EPSC and change ratio of the device under different pulse numbers, *A*_*n*_/*A*_*n−*1_ and *A_n_*/*A*_1_, of the *n* pulse to the first pulse. (I) Potentiation and depression of plasticity of synaptic devices. SDDP, spike-duration-dependent plasticity; SNDP, spike-number-dependent plasticity.

The synaptic device exhibits nonlinear conductance modulation characteristics analogous to those of biological synaptic transmission [33–36]. Systematic electrical performance characterization of the device was performed by applying gate voltage pulses to induce controlled proton migration and injection. This ionic modulation mechanism enables graded PSC responses spanning almost 3 orders of magnitude (Fig. 3A to C). Notably, the device demonstrates spiking voltage-dependent plasticity with ultralow operating voltages (−0.2 V).

The PSC exhibited precise tunability through systematic modulation electrical parameters. With an increase in the duration, the number, or the frequency of voltage spikes, the PSC of the synaptic device increases synchronously. Spike-duration-dependent plasticity (Fig. 3D and E and Fig. S1), spike-frequency-dependent plasticity (Fig. 3F and Fig. S2), and spike-number-dependent plasticity (Fig. 3G and H and Fig. S3) were emulated. These modulation capabilities demonstrate the device’s superior performance in neuromorphic information processing, which originates from precisely controlled ionic intercalation/release dynamics.

Furthermore, the PSC demonstrated duplex modulation capability through the application of opposite polarity pulses (−4 V/+1.5 V). The negative pulses (−4 V) induced synaptic potentiation, as evidenced by the paired-pulse facilitation effect, where the PSC exhibited interval-dependent variations under constant pulse stimulations (Fig. S4). Conversely, positive pulses (+1.5 V) triggered synaptic depression, enabling rapid reset of the PSC to its baseline potential (Fig. 3I).

### Mechanoelectronic coupling in the PEG synaptic device

Sensory information begins at skin receptors, which detect stimuli like touch or pain and convert them into electrical signals. These signals travel through peripheral nerves to the spinal cord and are relayed to the brain via the thalamus. Finally, the cerebral cortex processes the information, enabling conscious perception [37]. While previous neuromorphic implementations have replicated discrete sensory modalities (visual [38], tactile [39], and auditory [40]) through hybrid sensor–synapse architectures, the modular designs incur substantial energy penalties and integration limitations due to redundant signal transduction between physically separated components.

**Fig. 4.**
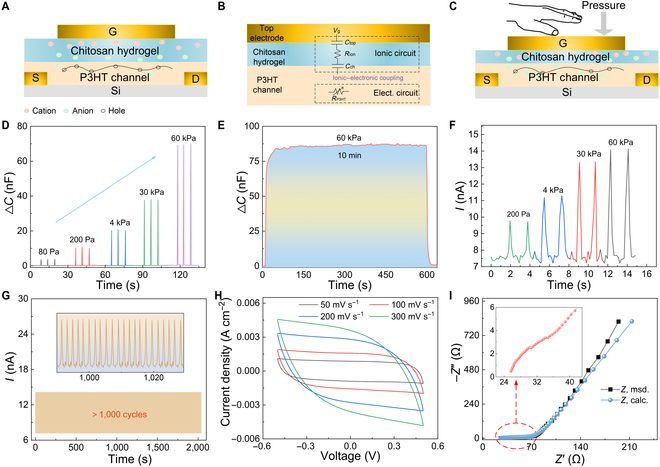
Mechanistic verification of sensor–computing integrated synaptic devices. (A) Ion distribution in sensor–computing integrated synaptic devices under *V*_g_ (*V*_g_ < 0). (B) Equivalent electrical model based on the ionic transport mechanism in the device. (C) Ionic distribution in sensor–computing integrated synaptic devices under *V*_g_ and pressure. (D) The capacitance variation of the electric double layer (EDL) under different pressures. (E) The capacitance variation of the EDL under stable pressure. (F) The current variation of the EDL under different pressures. (G) The current stability of the EDL. (H) Cyclic voltammetry (CV) test. (I) Electrochemical impedance spectroscopy (EIS) test.

Our monolithic design avoids these limitations by integrating tactile perception and synaptic computation in a monolithic device. The ions in the hydrogel were redistributed under gate voltage (−4 V), changing the electric double layer (EDL) at both gate/dielectric and dielectric/channel interfaces (Fig. 4A). The equivalent transport circuit of the device is demonstrated (Fig. 4B), where *C*_top_ is the capacitance between the gate electrode and the hydrogel, *R*_ion_ is the resistance of the chitosan hydrogel, and *C*_ch_ is the capacitance between the hydrogel and P3HT. Pressure induces a reduction in *R*_ion_, consequently modifying voltage distribution across circuit nodes. This redistribution drives *C*_ch_ variations, thereby inducing excitatory postsynaptic current (EPSC) modulation (Fig. 4C). While the chitosan hydrogel demonstrates inherent piezoelectric responses (Fig. S5), the pressure-induced piezoelectric potential (<15 mV) is much lower than the operational gate voltage (−4 V). This voltage disparity confirms that EPSC is mainly affected by the EDL mechanism rather than piezoelectric effects.

Systematic EDL analysis was performed to characterize the pressure-modulated capacitive behavior. The interfacial capacitance between the dielectric layer and the semiconductor channel displayed a monotonic increase with applied pressure (Fig. 4D), demonstrating exceptional operational stability with <2% capacitance drift during prolonged loading (10-min continuous pressure, Fig. 4E). Furthermore, to verify the stability of the contact between the chitosan hydrogel and the P3HT layer, we compared the height profile from the chitosan hydrogel surface to the P3HT surface before and after pressing using a profilometer (Fig. S6). After compression, the height of the central compression area of the hydrogel decreased by 0.645 μm, while the height of the edge area of the hydrogel decreased by 0.097 μm. This phenomenon indicates that the hydrogel did not warp after compression and maintained stable contact with the P3HT layer.

The pressure–current response was systematically investigated through dynamic compression testing (200 Pa to 60 kPa). Real-time current monitoring revealed a pressure-proportional current enhancement (Fig. 4F) with exceptional operational stability (<1% signal deviation during prolonged loading, 2,000 s, Fig. 4G).

Electrochemical analyses were performed to elucidate the underlying ionic dynamics. Cyclic voltammetry displayed quasi-rectangular profiles (scan rate: 50 mV/s; Fig. 4H), confirming dominant EDL formation without faradaic reactions. Electrochemical impedance spectroscopy further resolved the ionic diffusion process (Fig. 4I), indicative of charge-transfer resistance at the electrolyte/channel interface.

### Affective tactile encoding for socially intelligent interfaces

**Fig. 5.**
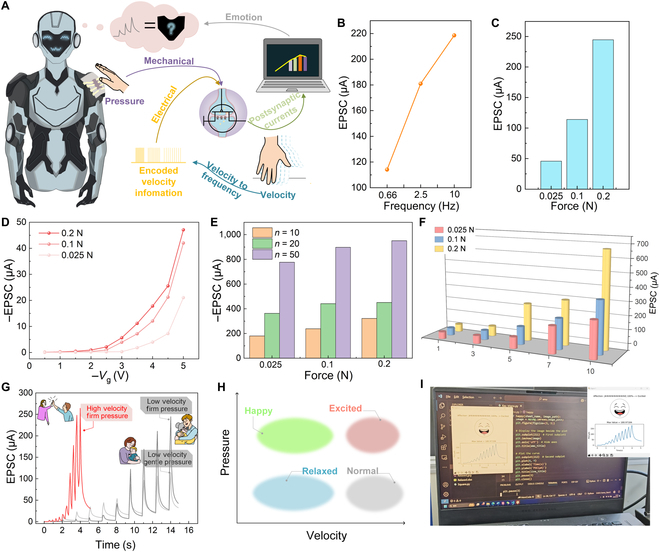
Sensor–computing integrated device for emotional discrimination in robots. (A) Schematic diagram of force–electric dual-mode coding for sensor–computing integrated devices. (B) EPSC under different frequencies of *V*_g_ (same pressure). (C) EPSC under different pressures (same frequency of *V*_g_). (D) EPSC under different pressures and *V*_g_ values. (E) EPSC under different pressures and spike numbers. (F) EPSC under different pressures and durations. (G) EPSC under 3 actions: clapping (fast velocity and strong pressure), patting (slow velocity and strong pressure), and rocking (slow velocity and low pressure). (H) The emotions associated with different tactile behaviors. (I) EPSC was read for emotion classification.

Current tactile interface technologies predominantly focus on discriminative sensing through physical parameter quantification, e.g., pressure mapping and texture recognition [41,42]. Emerging evidence from psychosensory studies reveals the critical role of affective tactile processing mediated by CT afferents, which are low-threshold mechanoreceptors. These CT-mediated signals propagate through the posterior insular cortex to generate emotional valence, forming the neurobiological basis for touch-induced social communication. Leveraging this mechanism, we demonstrate the application of the PEG synaptic in emotion discrimination capable of decoding 3 discrete emotional states, namely, happiness, calmness, and excitement, through spatiotemporal stimulus coding (Fig. 5A).

To systematically investigate the multimodal plasticity of the PEG synaptic device, we conducted comprehensive EPSC testing across 5 stimulation dimensions (Fig. 5B to F): EPSC under different frequencies of *V*_g_ with the same pressure (Fig. 5B), EPSC under different pressures with the same frequency of *V*_g_ (Fig. 5C), EPSC under different pressures and *V*_g_ values (Fig. 5D), EPSC under different pressures and spike numbers (Fig. 5E), and EPSC under different pressures and durations (Fig. 5F). These experiments confirm monotonic EPSC enhancement with increasing stimulation intensity across all modalities.

The emotional classification criterion (Fig. 5H and Fig. S2) was established through psychophysical analysis of human social touch perception [43–45], where controlled experiments with volunteer participants validated the correlations between specific tactile patterns and emotional responses. Our neuromorphic system implements these associations by converting the tactile parameters into corresponding EPSC amplitude thresholds, enabling matching of spatiotemporal tactile inputs to discrete emotional states.

As a proof of concept, we established 3 tactile–emotional mapping paradigms: (a) low velocity (0.66 Hz) and gentle pressure (0.025 N), mimicking maternal tactile interactions; (b) low velocity (0.66 Hz) and firm pressure (0.2 N), simulating friendly embraces; and (c) high velocity (2.5 Hz) and firm pressure (0.2 N), replicating celebratory high fives (Fig. 5G). The emotional states were quantified based on the amplitude of the EPSC recorded after 10 consecutive stimuli. Specifically, the peak current value of the final spike in the EPSC sequence was extracted and categorized into 3 discrete emotional states using predefined threshold ranges: calmness, <150 μA; happiness, 150 to 250 μA; and excitement, >250 μA.

EPSC measurements under 3 stimulation modes revealed distinct neural responses: (a) maternal-type stimuli elicited baseline EPSC amplitudes of 114.71 μA, inducing parasympathetic activation associated with calmness; (b) embrace-mimetic interactions generated intermediate EPSC signals (244.78 μA), correlating with positive valence responses characteristic of happiness; and (c) high-five simulations produced maximal EPSC outputs (264.60 μA), triggering sympathetic arousal indicative of excitement (Fig. 5H).

The PEG synaptic device generated differentiated EPSC corresponding to specific tactile patterns, which were computationally classified into discrete emotional states through amplitude threshold analysis. Fig. 5I shows the emotion recognition results of the computer. This neuromorphic cognitive architecture, implementing tactile–emotional associations through spatiotemporal EPSC encoding, establishes a technological foundation for next-generation human–robot interaction platforms requiring affective intelligence.

## Conclusion

We fabricated a neuromorphic device capable of spatiotemporal tactile discrimination (pressure and motion perception) and neuro-inspired information processing in the monolithic structure. The device is operable by applied pressure and applied voltage and synergistic electrical–mechanical encoding through the coupling of voltage-induced ion migration and pressure-enhanced ion injection. Based on this neuromorphic tactile device, we constructed an affective tactile recognition system capable of classifying 3 discrete emotional states (calmness, happiness, and excitement). The system demonstrates important potential for transformative applications in social robotics, establishing a new paradigm for bioinspired tactile sensing and bridging the gap between neuromorphic engineering and affective computing through its unique combination of ionic–electronic coupling and spatiotemporal encoding capabilities.

## Data Availability

All data are available in the article or the supplementary materials.
