# Supplementary figures and images for: An Integrated Monolithic Synaptic Device for C-Tactile Afferent Perception and Robot Emotional Interaction
Source: Cyborg Bionic Syst. 2025 Aug 19;6:0367. doi: 10.34133/cbsystems.0367 (PMC12364544; doi:10.34133/cbsystems.0367)

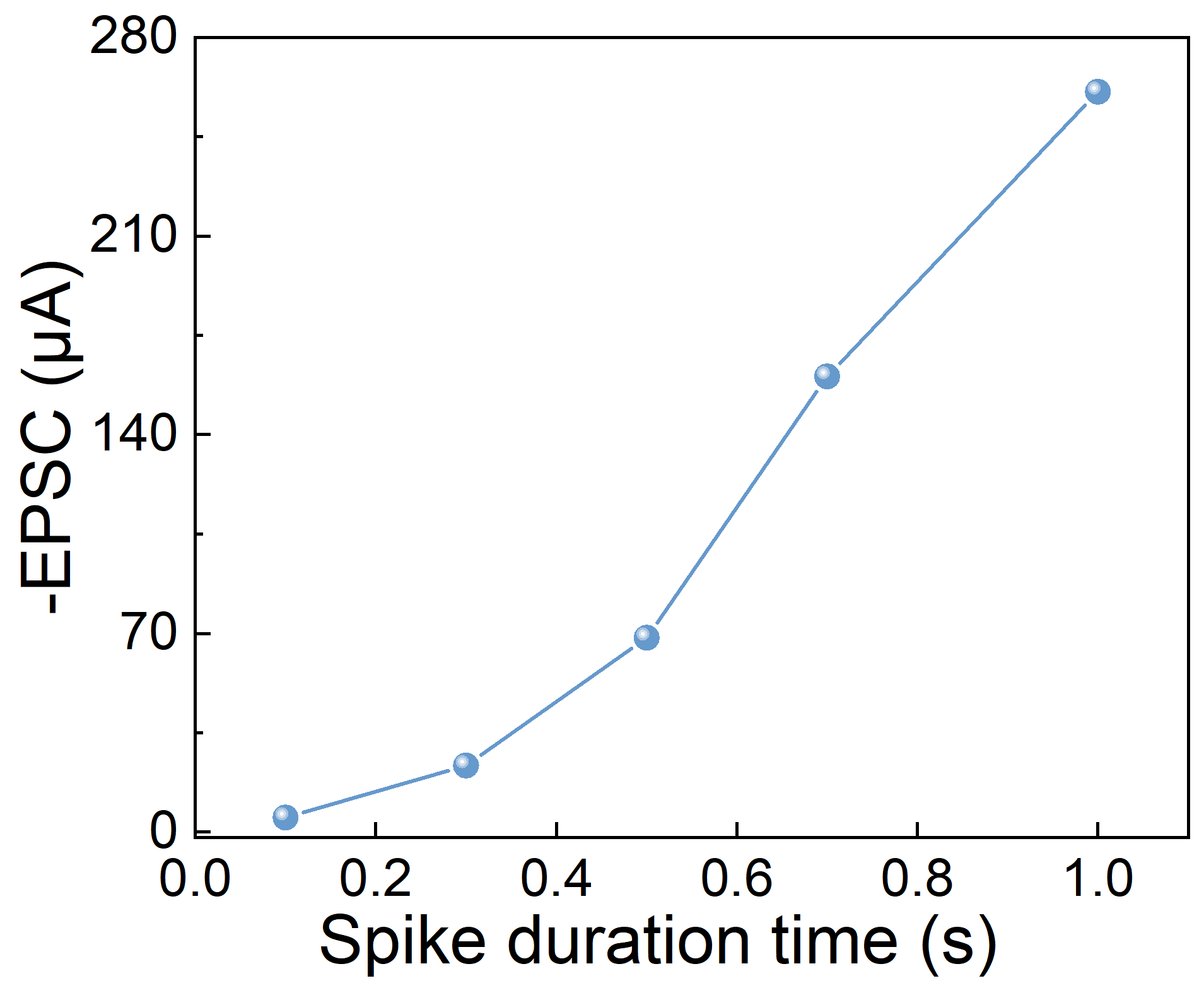

Supplement: Supplementary 1 — Figs. S1 to S6 Tables S1 and S2 [file cbsystems.0367.f1.zip › S1.tif]

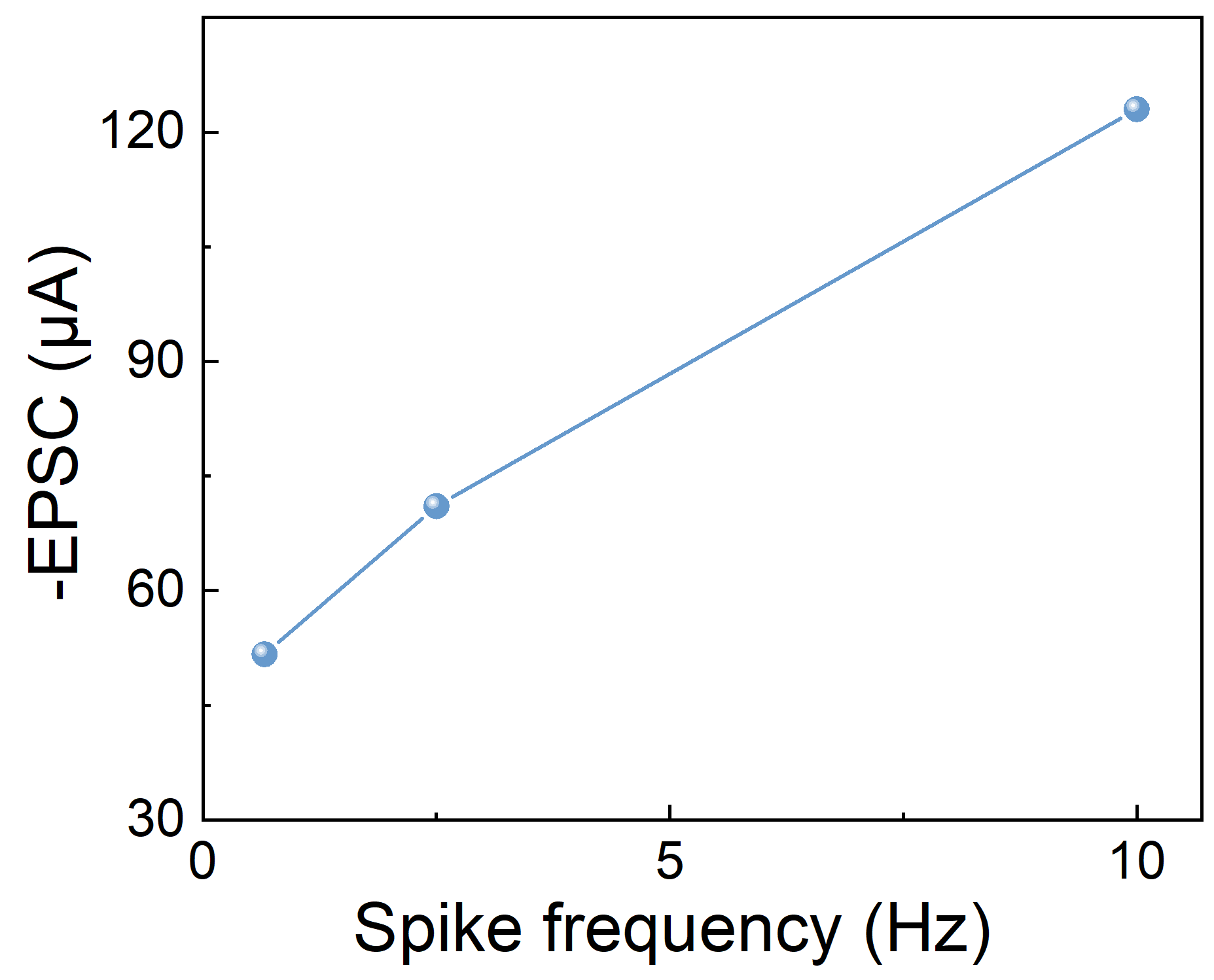

Supplement: Supplementary 1 — Figs. S1 to S6 Tables S1 and S2 [file cbsystems.0367.f1.zip › S2.tif]

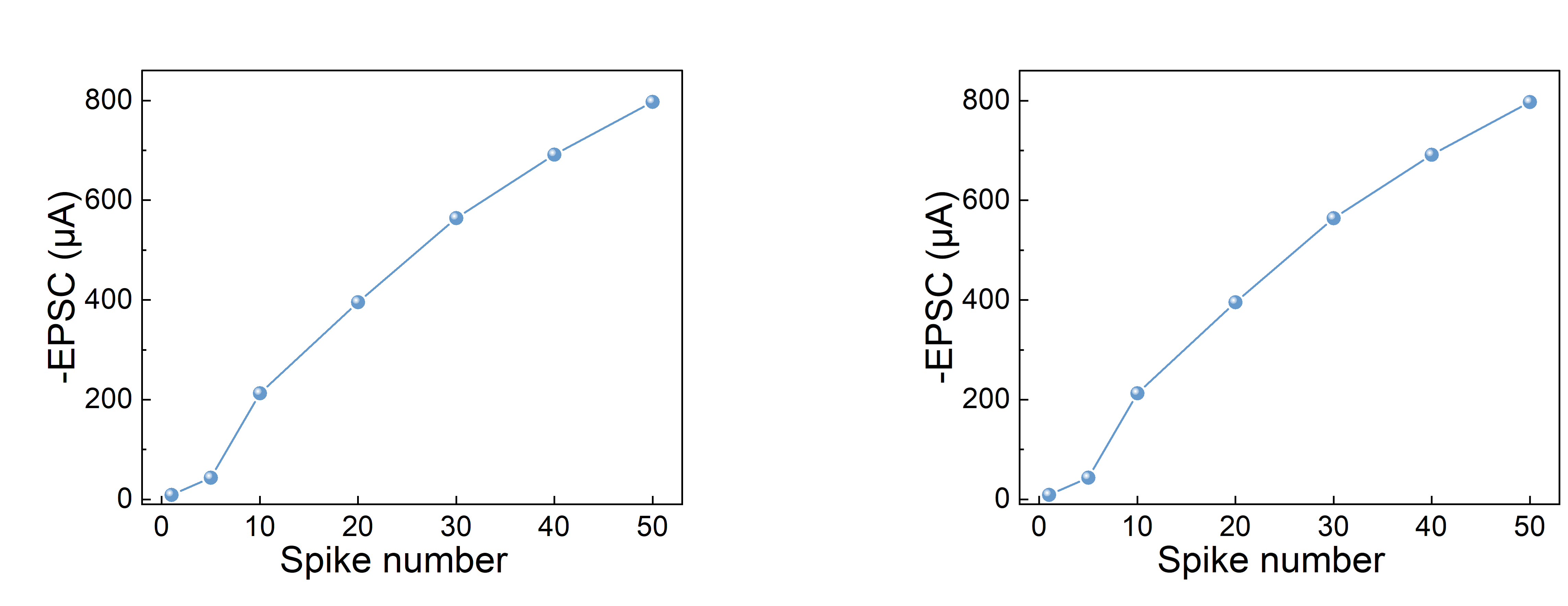

Supplement: Supplementary 1 — Figs. S1 to S6 Tables S1 and S2 [file cbsystems.0367.f1.zip › S3.tif]

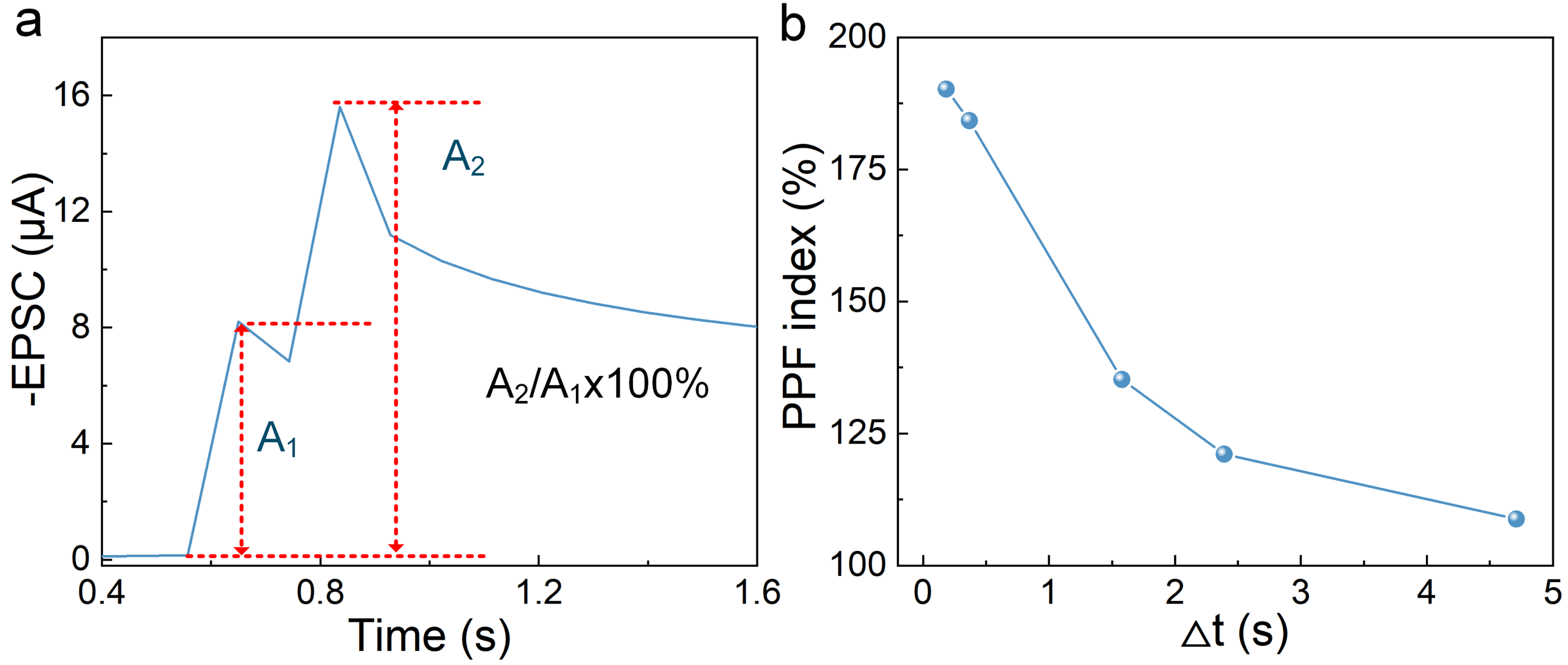

Supplement: Supplementary 1 — Figs. S1 to S6 Tables S1 and S2 [file cbsystems.0367.f1.zip › S4.tif]

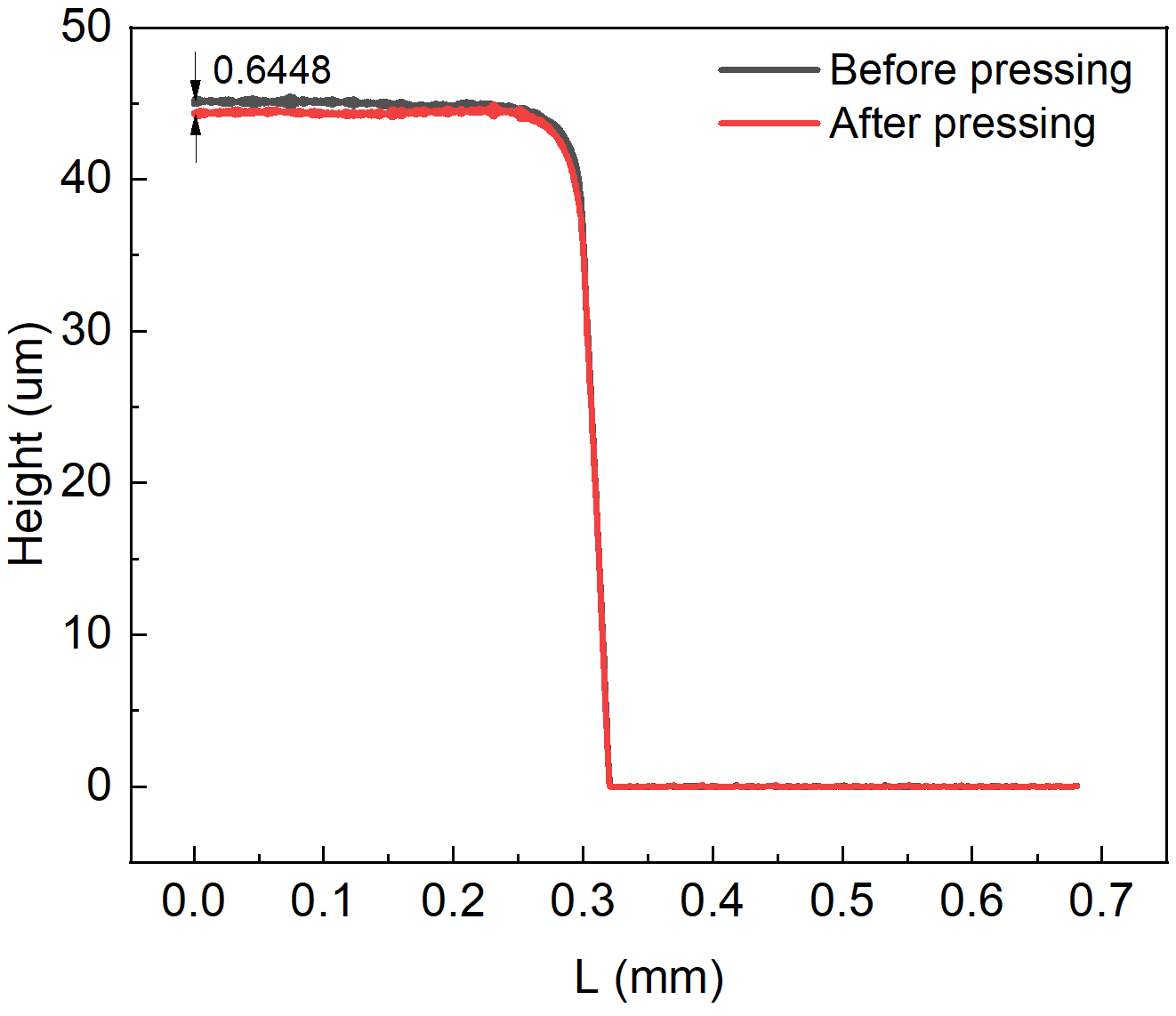

Supplement: Supplementary 1 — Figs. S1 to S6 Tables S1 and S2 [file cbsystems.0367.f1.zip › S5-.tif]
