# Supplementary material for: An Integrated Monolithic Synaptic Device for C-Tactile Afferent Perception and Robot Emotional Interaction
Source: Cyborg Bionic Syst. 2025 Aug 19;6:0367. doi: 10.34133/cbsystems.0367 (PMC12364544; doi:10.34133/cbsystems.0367)
Supplement: Supplementary 1 — Figs. S1 to S6 Tables S1 and S2 [file cbsystems.0367.f1.zip › Figure captions.doc]

**Fig. S1.** SDDP maximum of neuromorphic devices.

**Fig. S2.** SFDP maximum of neuromorphic devices.

**Fig. S3.** SNDP maximum of neuromorphic devices.

**Fig. S4.** Relationship between pulse time interval and PPF index (*A_2_*/*A_1_*×100%), consecutive pulses (-4 V, 10 ms) to simulate PPF behavior (*A_1_* and *A_2_* are the first and second response peaks respectively).

**Fig. S5.** The interface changes before and after pressing.

**Fig. S6.** the photograph of the device and interfacial morphology.
